# Supplementary figures and images for: Dysregulated hemolysin liberates bacterial outer membrane vesicles for cytosolic lipopolysaccharide sensing
Source: PLoS Pathog. 2018 Aug 23;14(8):e1007240. doi: 10.1371/journal.ppat.1007240 (PMC6124777; doi:10.1371/journal.ppat.1007240)

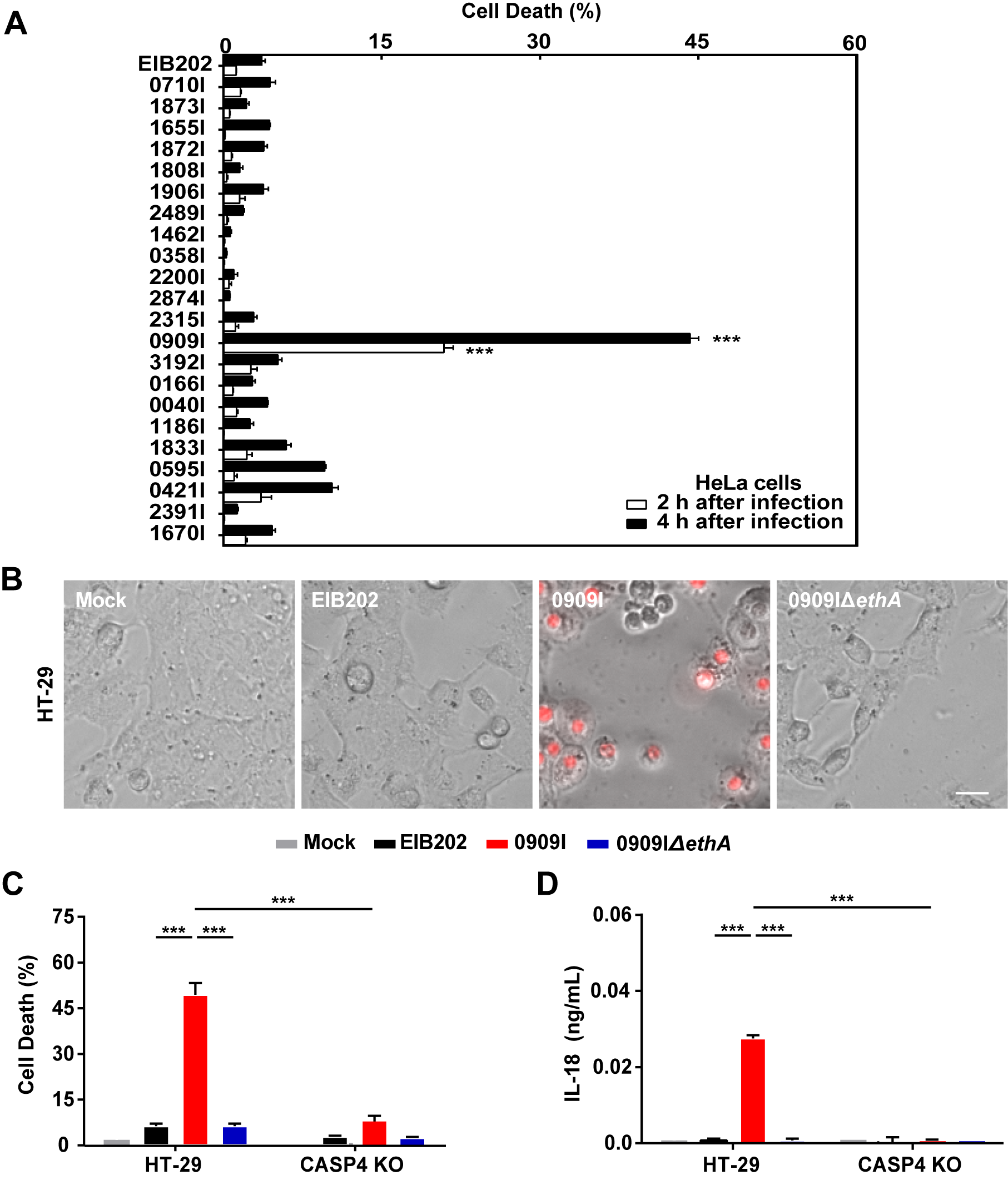

Supplement: S1 Fig — (A) LDH release detected in wild-type HeLa cells infected with the gene-defined mutant library of E. tarda at MOI = 100 for the indicated time periods, only partial data were showed here. (B) Cell morphology with PI staining of HT-29 cells infected with indicated E. tarda strains (MOI = 25, 4 hpi), scale = 20 μm. (C-D) LDH release (C) and IL-18 secretion (D) detected in wild-type and Caspase-4-/- HT-29 cells infected with indicated E. tarda strains (MOI = 25, 4 hpi). Graphs show the mean and s.e.m. of triplicate wells and are representative of three independent experiments. *p < 0.05, **p < 0.01, ***p < 0.001; NS, not significant (two-tailed t-test). (TIF) [file ppat.1007240.s001.tif]

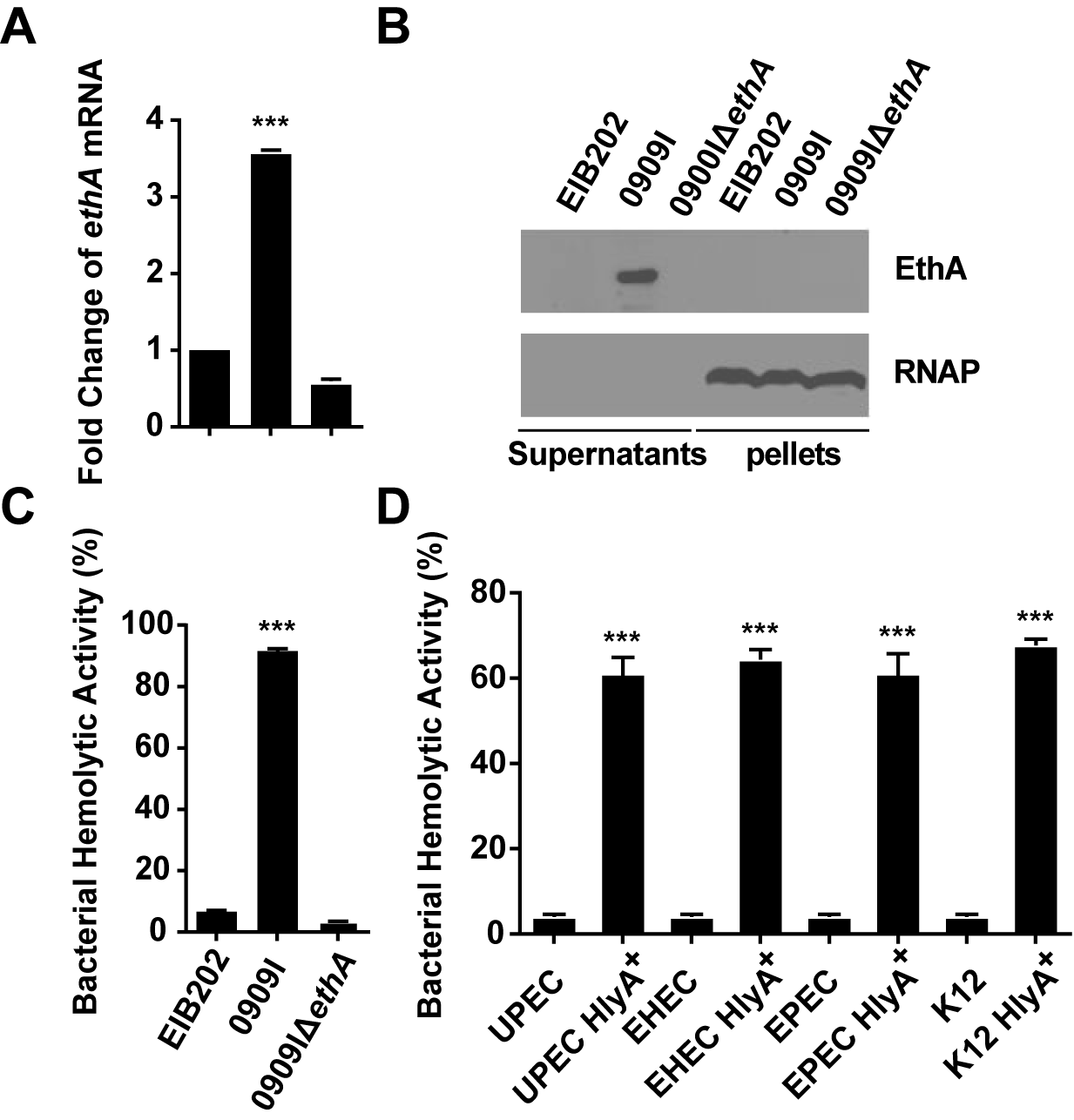

Supplement: S2 Fig — (A) Quantitative PCR for ethA mRNA in E. tarda strains. (B) Immunoblots for EthA in the pellets and supernatants of E. tarda cultures. (C-D) Assay of hemolytic activity in the indicated E. tarda (C) or E. coli strains (D). Graphs show the mean and s.e.m. of triplicate wells and are representative of three (A, C and D) independent experiments. *p < 0.05, **p < 0.01, ***p < 0.001; NS, not significant (two-tailed t-test). (TIF) [file ppat.1007240.s002.tif]

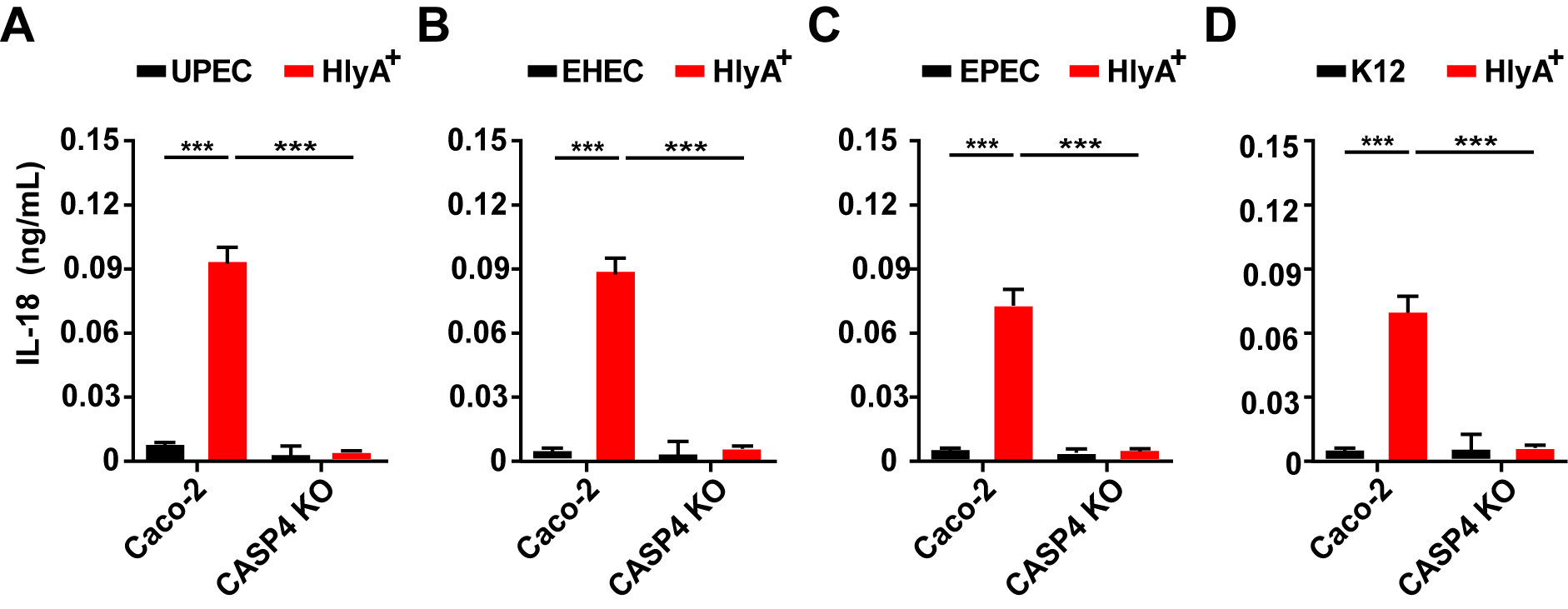

Supplement: S3 Fig — (A-D) IL-18 secretion detected in wild-type and Caspase-4-/- Caco-2 cells infected with the indicated E. coli strains (MOI = 50, 4 hpi). Graphs show the mean and s.e.m. of triplicate wells and are representative of three independent experiments. *p < 0.05, **p < 0.01, ***p < 0.001; NS, not significant (two-tailed t-test). (TIF) [file ppat.1007240.s003.tif]

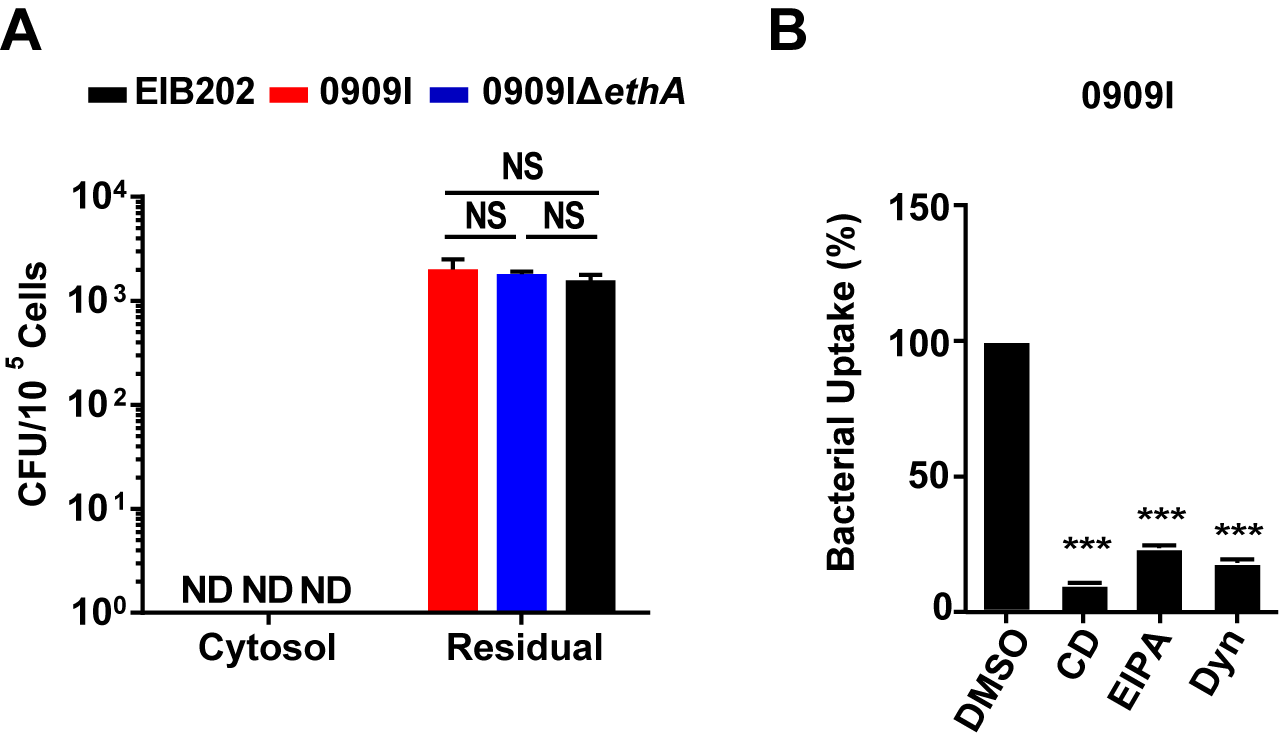

Supplement: S4 Fig — (A) Bacterial count by agar plating in the cell pellets after treatment with 300 μg/mL gentamicin for 1 h to kill extracellular bacteria, or in the cytosolic fraction extracted by digitonin fractionation, both from Caspase-4-/- Caco-2 cells infected with the indicated strains (MOI = 25, 4 hpi). (B) Bacterial count by agar plating in the cell pellets from Caspase-4-/- Caco-2 cells incubated with indicated E. tarda 0909I (MOI = 25, 4 hpi), following treatment with 300 μg/mL gentamicin for 1 h to kill extracellular bacteria. Graphs show the mean and s.e.m. of triplicate wells and are representative of three independent experiments. *p < 0.05, **p < 0.01, ***p < 0.001; NS, not significant (two-tailed t-test). (TIF) [file ppat.1007240.s004.tif]

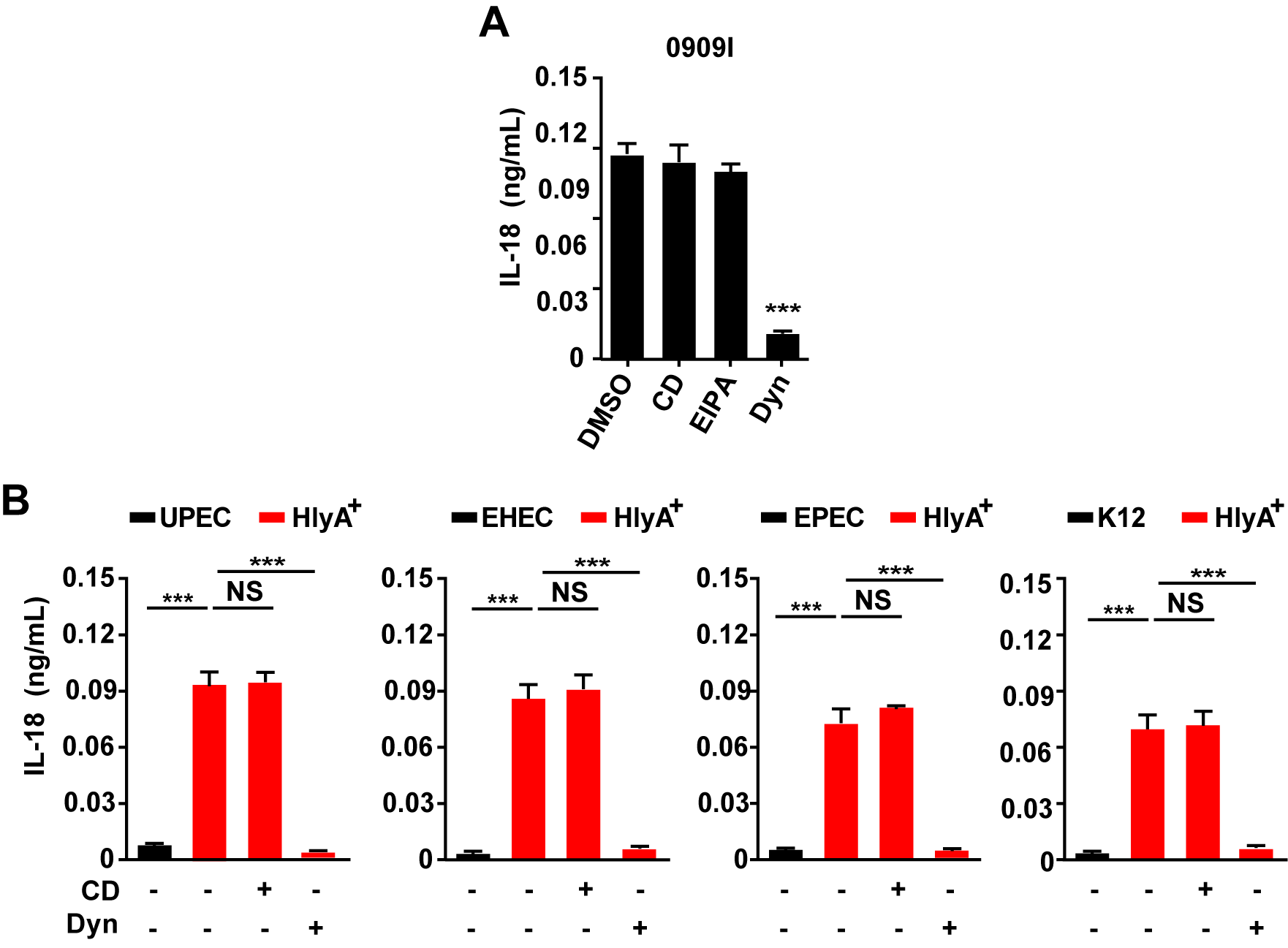

Supplement: S5 Fig — (A-B) IL-18 secretion in Caco-2 cells infected by E. tarda 0909I (MOI = 25, 4 hpi) (A) or the indicated E. coli strains (MOI = 50, 4 hpi) (B), in the presence of CD (10 μM), EIPA (30 μM), Dyn (80 μM), or not. Graphs show the mean and s.e.m. of triplicate wells and are representative of three independent experiments. *p < 0.05, **p < 0.01, ***p < 0.001; NS, not significant (two-tailed t-test). (TIF) [file ppat.1007240.s005.tif]

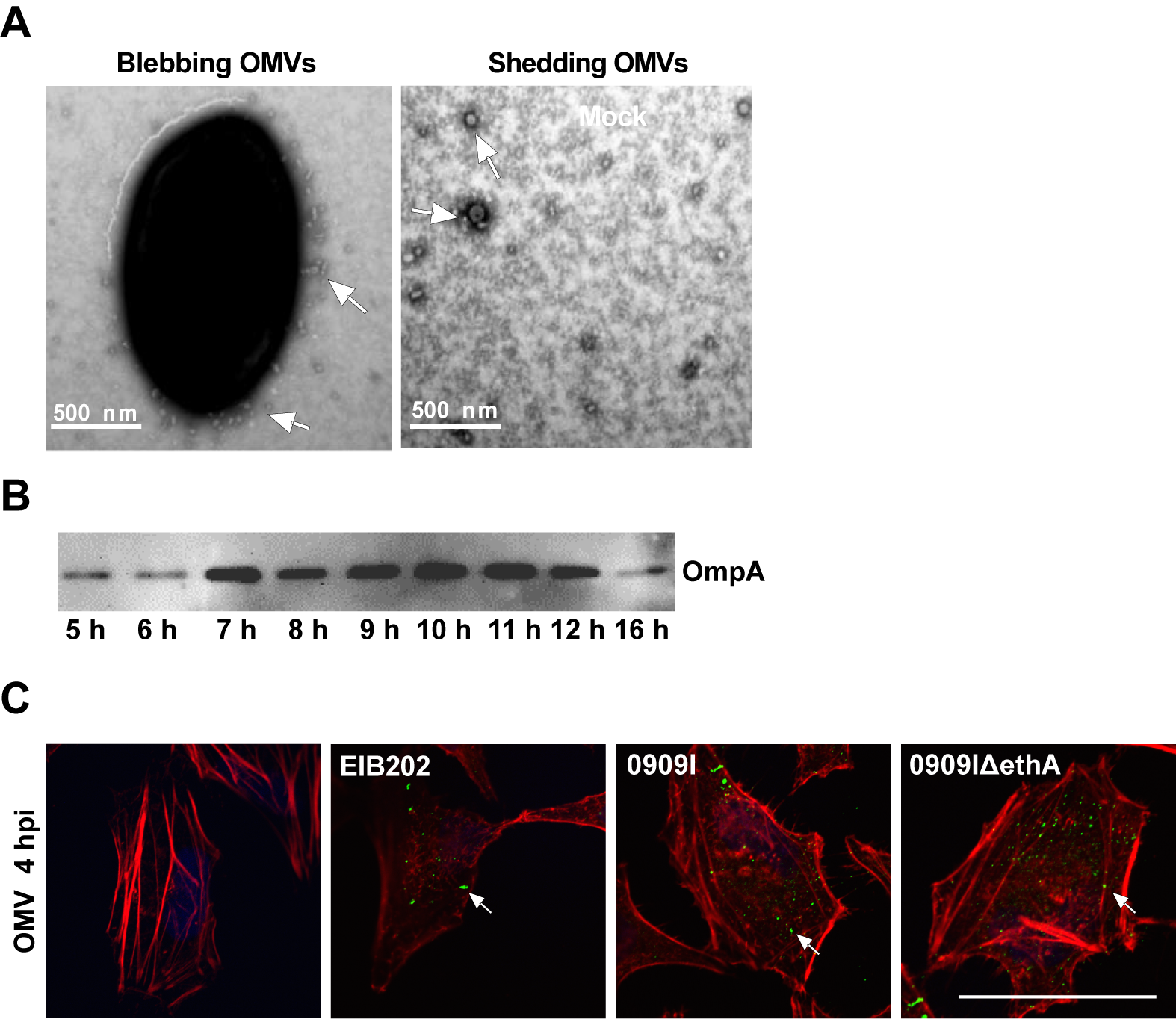

Supplement: S6 Fig — (A) Observation of OMVs in the culture of E. tarda EIB202 at 10 h post-inoculation under transmission electron microscope, scale = 500 nm, the white arrows indicate the OMVs. (B) Immunoblots for OmpA, a stably expressed outer membrane protein, in the concentrated supernatants of E. tarda EIB202 to roughly quantify OMVs at indicated time periods. (C) Immunostaining for intracellular OMV specks using anti-OmpA antibody in HeLa cells incubated with the indicated E. tarda OMVs (20 μg/1 × 105 cells, 4 h), Scale = 20 μm. (TIF) [file ppat.1007240.s006.tif]

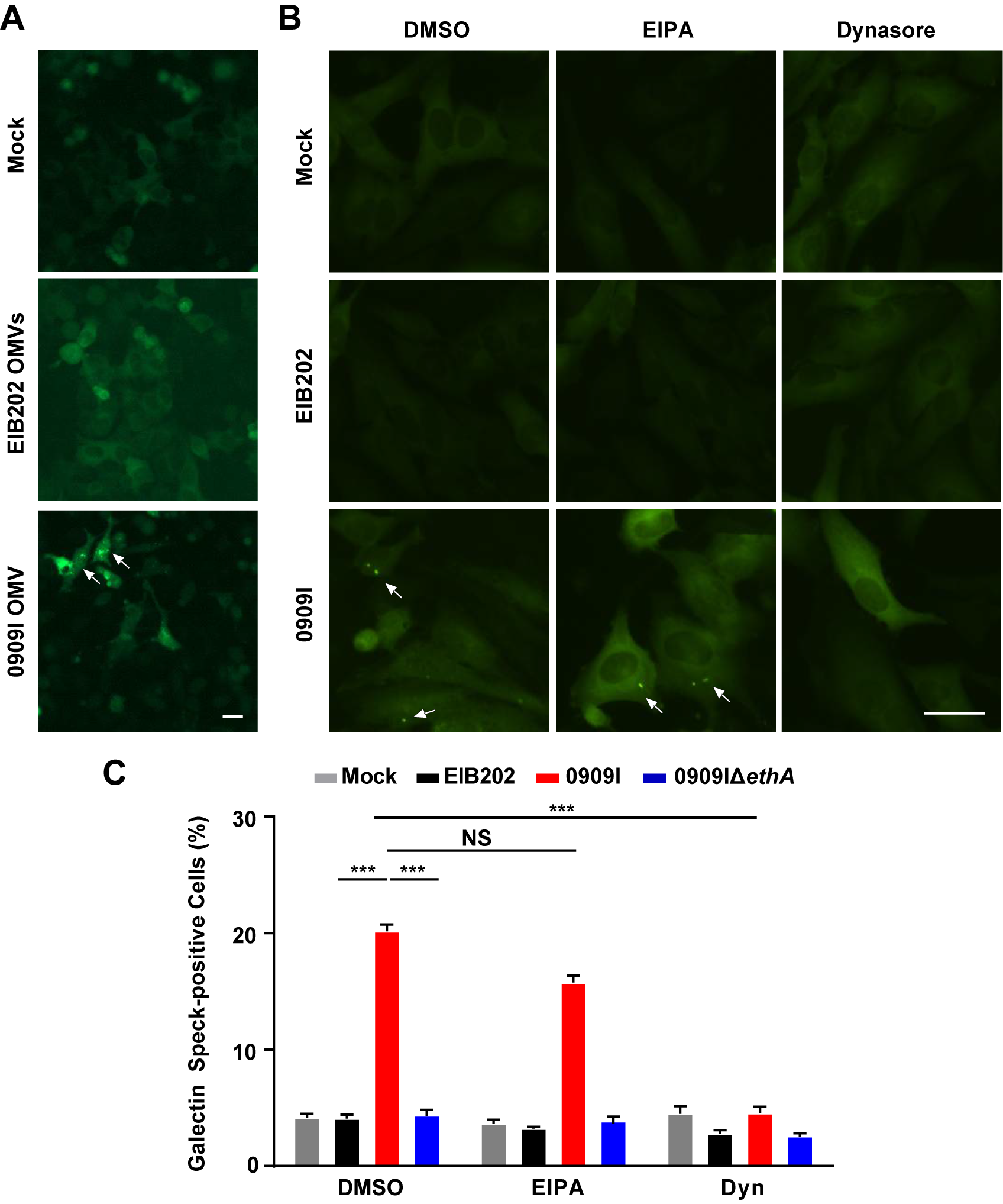

Supplement: S7 Fig — (A-B) Formation of galectin specks within HeLa cells expressing GFP-tagged gelectin-3, incubated with E. tarda OMVs (50 μg, 1 × 10^5^ cells, 16 h) (A), or infected with E. tarda stains (MOI = 25, 4 hpi) pretreating with EIPA (30 μM), Dyn (80 μM), or not (B), scale = 20 μm, the white arrows indicate the galectin specks within cells. (C) Quantification of galectin speck-positive cells in the infected cells as described in (B), the percentage of cells containing galectin specks was calculated for at least 500 cells. Graphs show the mean and s.e.m. of triplicate wells and are representative of three independent experiments. *p < 0.05, **p < 0.01, ***p < 0.001; NS, not significant (two-tailed t-test). (TIF) [file ppat.1007240.s007.tif]

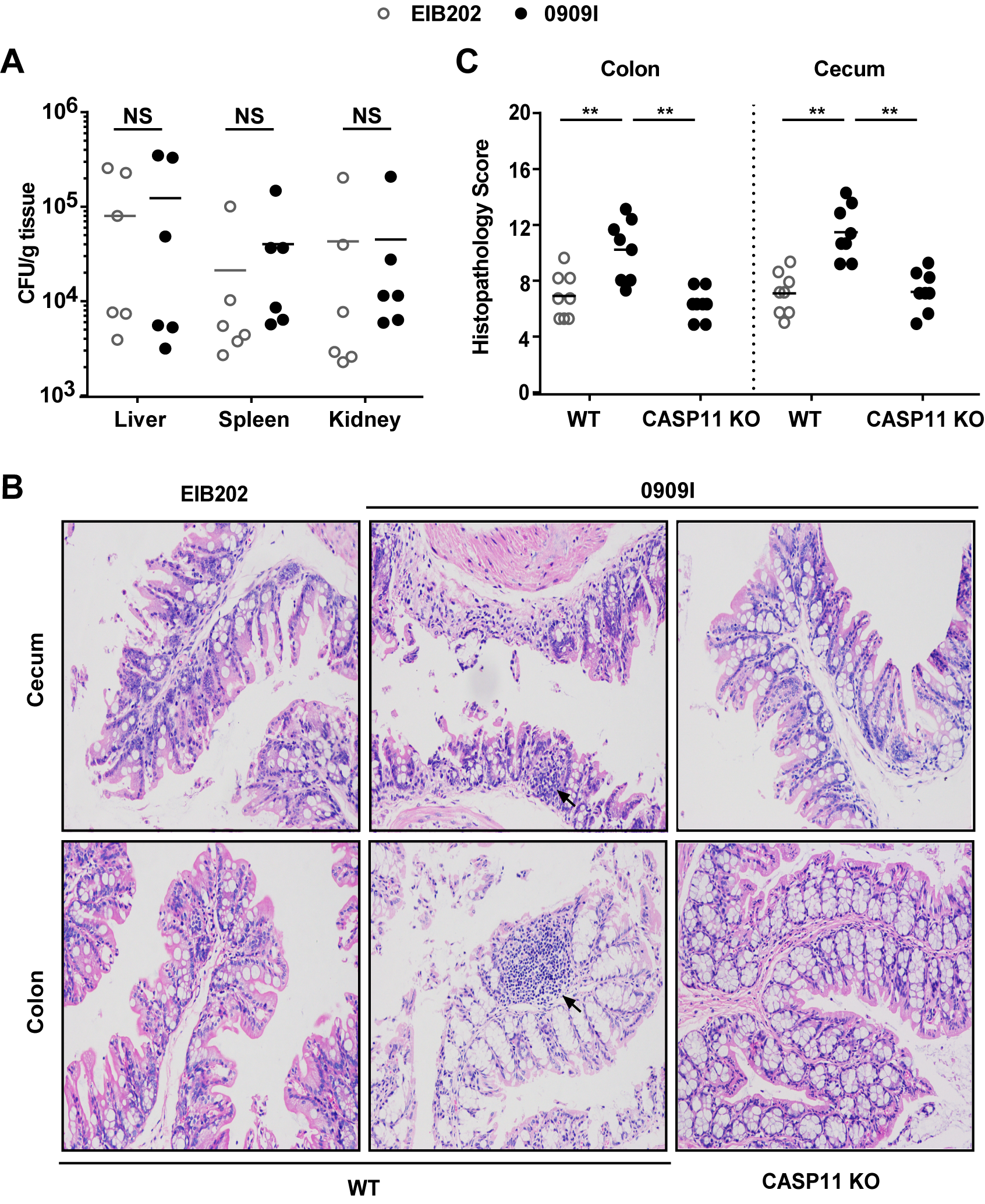

Supplement: S8 Fig — (A) Bacterial counting by agar plating in the liver, spleen, and kidney of wild-type mice orally-infected by EIB202 or 0909I (5 × 10^7^ cfu/g) at 24 hpi. (B) H&E staining of the colon and caecum sections from the mice described in A, magnification = 200 ×, the black arrows indicate the inflammatory focal infiltration (IFI). (C) Histological scores of the gut sections in (B). Graphs depict 6–8 mice per genotype and are representative of two independent experiments. *p < 0.05, **p < 0.01, ***p < 0.001; NS, not significant (one-way ANOVA). (TIF) [file ppat.1007240.s008.tif]
